# The effect of particle size and amount of inoculum on fungal treatment of wheat straw and wood chips

**DOI:** 10.1186/s40104-016-0098-4

**Published:** 2016-07-13

**Authors:** Sandra J. A. van Kuijk, Anton S. M. Sonnenberg, Johan J. P. Baars, Wouter H. Hendriks, John W. Cone

**Affiliations:** Animal Nutrition Group, Wageningen University, De Elst 1, 6708WD Wageningen, The Netherlands; Plant Breeding, Wageningen University, Droevendaalsesteeg 1, 6708PB Wageningen, The Netherlands

**Keywords:** Amount of inoculum, Fungal treatment, In vitro rumen degradability, Lignin degradation, Lignocellulosic biomass, Particle size

## Abstract

**Background:**

The aim of this study was to optimize the fungal treatment of lignocellulosic biomass by stimulating the colonization. Wheat straw and wood chips were treated with *Ceriporiopsis subvermispora* and *Lentinula edodes* with various amounts of colonized millet grains (0.5, 1.5 or 3.0 % per g of wet weight of substrate) added to the substrates. Also, wheat straw and wood chips were chopped to either 0.5 or 2 cm. Effectiveness of the fungal treatment after 0, 2, 4, 6, or 8 wk of incubation was determined by changes in chemical composition, in vitro gas production (IVGP) as a measure for rumen degradability, and ergosterol content as a measure of fungal biomass.

**Results:**

Incomplete colonization was observed for *C. subvermispora* treated wheat straw and *L. edodes* treated wood chips. The different particle sizes and amounts of inoculum tested, had no significant effects on the chemical composition and the IVGP of *C. subvermispora* treated wood chips. Particle size did influence *L. edodes* treatment of wheat straw. The *L. edodes* treatment of 2 cm wheat straw resulted in a more selective delignification and a higher IVGP than the smaller particles. Addition of 1.5 % or 3 % *L. edodes* inoculum to wheat straw resulted in more selective delignification and a higher IVGP than addition of 0.5 % inoculum.

**Conclusion:**

Particle size and amount of inoculum did not have an effect on *C. subvermispora* treatment of wood chips. At least 1.5 % *L. edodes* colonized millet grains should be added to 2 cm wheat straw to result in an increased IVGP and acid detergent lignin (ADL) degradation.

## Background

Cellulose is one of the most abundant carbohydrates in the world and is, next to starch, an important carbon source in a ruminants diet. In plant cell walls, cellulose is bound to hemicellulose and lignin in the lignocellulosic complex. Lignin is a polymer that is difficult to degrade, and is only degradable under aerobic conditions [1]. In the anaerobic rumen, lignin is hardly affected and as a result, the lignocellulosic complex has a limited degradability [2]. As a result, cellulose and hemicellulose in this complex have a limited availability to the rumen microbes. To increase this availability of cellulose, and thus rumen degradability, lignin should be removed before feeding lignocellulosic biomass to ruminants. Lignin removal can be achieved using several pre-treatment methods [3, 4], for example specific lignin degrading fungal or bacterial species [5] These biological pre-treatments are a relatively cheap and environmental friendly alternatives for chemical and physical pre-treatments [6]. Although some bacterial species show lignin degrading activities, fungal systems are more oxidatively powerful compared to bacterial systems [7]. Furthermore lignin is a complex substrate that is known to require maximum oxidative enzymes to its degradation. That is best done in a solid state fermentation for which fungi are more suited. Bacteria flourish better in a watery environment [7]. In the current study, white rot fungi were selected based on their selectivity for lignin degradation. Pre-treatments using selective lignin degrading fungi, such as *Ceriporiopsis subvermispora* and *Lentinula edodes,* were proven to increase the in vitro rumen degradability as a consequence of increased cellulose availability of wheat straw and sugarcane bagasse [8, 9]. Also, in vivo studies reported in the scientific literature show an increased digestibility of fungal treated biomass; e.g. treatment with *C. subvermispora* increased digestibility of bamboo in sheep [10], treatment with *Ganoderma* spp. increased the digestibility of wheat straw in goats [11] and a treatment with *Pleurotus* spp. increased the digestibility, however only a maximum of 17 % dietary inclusion rate of fungal treated material was accepted by cows [12].

The time needed to obtain maximum cellulose availability to increase in vitro rumen degradability was 6 to 12 wk [8, 9, 13]. Although the substrates after the fungal pre-treatment were enriched in cellulose, hemicellulose was partially used by the fungi as lignin was degraded. This relatively long treatment time and consumption of part of the carbohydrate fraction are major drawbacks and optimization of both is needed to make this method competitive with current chemical and physical pre-treatments.

Fungal pre-treatment starts with the inoculation of the substrate. In scientific literature, inoculation for *C. subvermispora* or *L. edodes* treatments has been done using agar plugs [14–16] or using liquid medium [17–19]. However, in the commercial mushroom production process spawn is produced from grains [20]. Grain based spawn can be produced on a large scale and is easy to mix through the substrate. Previous studies have used spawn made from wheat, millet or sorghum grains [8–10, 13, 21]. Compared to other grains, sorghum grains are relatively large, meaning less inoculation points per g of inoculum added. The use of a smaller grain, like millet, would increase the amount of inoculation points per g of inoculum added. The latter can initiate a faster colonization of the lignocellulose biomass [22].

Rapid and complete colonization of the lignocellulosic material is key for a competitive fungal pre-treatment. During colonization, the fungus starts degrading the outer layer of the material before reaching the inside [23]. By decreasing the particle size of the substrate, the surface to volume ratio is increased. The latter will result in more contact points on the surface and reach the inner part of the material faster [22].

The aim of this study was to optimize the colonization conditions of the fungal pre-treatment to obtain the most selective delignification, with minimal carbohydrate degradation and a high in vitro rumen degradability. Based on the work of Tuyen et al. [8], *C. subvermispora* and *L. edodes* were chosen as selective lignin degraders. Wheat straw and wood chips were selected for the fungal treatment, because of their high content of lignocellulose, geographic availability and the fact that they are widely studied. Two different particle sizes, 0.5 and 2 cm length, of both substrates were used with different amounts of inoculum (colonized millet) added.

## Methods

### Fungal strains

*Ceriporiopsis subvermispora* (strain MES 13094) and *Lentinula edodes* (strain MES 11910) were initially cultured on malt extract (10 g malt extract and 17.5 g agar per L, Oxoid LP0039, Thermo Scientific, Hampshire, UK) agar until it was almost fully colonized. Agar pieces (approximately 1 cm^2^) were added to sterilized millet grains. Inoculated millet grains were incubated at 24 °C until full colonization, which occurred 5 wk after the start of incubation. Fully colonized grains were used as spawn to inoculate the substrates.

### Substrates

Wheat straw and wood chips (oak) were used as substrates. Both substrates were chopped to particles with average sizes of 0.5 cm or 2 cm. The substrates were submerged in water for 3 d after which the excess water was removed by draining through nets and the substrates divided over 1.2 L autoclavable polypropylene containers and covered with a lid containing a filter (model TP1200 + TPD1200 XXL Combiness, Nazareth, Belgium) through which air can pass. To each container 200 g of wet substrate was added and, since the water holding capacity of both substrates was different, this represented approximately 100 and 50 g dry matter of wood chips and wheat straw, respectively. The containers with substrate were sterilized for 1 h at 121 °C. The sterilized substrates were kept at room temperature until further use. Autoclaved, uninoculated samples were taken to serve as control.

### Inoculation

Colonized millet grains were added to each container in a concentration of either 0.5, 1.5 or 3 % of the weight of the wet substrate. The content of the containers were mixed under aseptic conditions to divide the inoculum equally over the substrate. All conditions were tested in triplicate, in which each sample started with 100 g dry matter (in case of wheat straw, divided over two containers). Samples were incubated for 2, 4, 6 and 8 wk in a climate controlled chamber at 24 °C and 70 % relative humidity. After incubation, the two containers of wheat straw were pooled to represent one sample of 100 g dry. Part of each sample (approximately 10 %) was freeze-dried for ergosterol measurements and the remaining part of the sample was air-dried at 70 °C for 1 wk to be used for chemical analyses and in vitro gas production measurements. The dried wheat straw was ground to 1 mm using a Peppink 100 AN cross beater mill (Peppink, Deventer, The Netherlands). The dried wood chips were finely ground over a 1 mm sieve using a Retch ZM100 centrifugal mill (Retsch, Haan, Germany) to obtain a homogenous sample.

The remaining colonized and uninoculated millet grains that were not used for inoculation were used for further analysis. Approximately 10 % of each sample was freeze-dried, and the remaining part was air-dried at 70 °C for 1 wk. The dried millet was ground to 1 mm using a Peppink 100 AN cross beater mill (Peppink, Deventer, The Netherlands).

### In vitro gas production technique

In vitro gas production (IVGP) was measured for all samples by the IVGP technique, according to Cone et al. [24]. In summary, 60 mL of buffered rumen fluid, collected from non-lactating cows, was added to 0.5 g air-dried material. Incubations were done in shaking water baths at 39 °C. Gas production was measured continuously for 72 h. Results were expressed as mL gas produced after 72 h per g organic matter (OM).

### Chemical analysis

Fiber analysis was performed according to Van Soest et al. [25], using an Ankom fiber analyser 2000 (ANKOM Technology, Macedon, New York, USA). The hemicellulose content was calculated as the difference between neutral detergent fiber (NDF) and acid detergent fiber (ADF) contents. The cellulose content was calculated as the difference between ADF and acid detergent lignin (ADL) contents.

For dry matter (DM) determination air-dried material was dried for 4 h at 103 °C. Ash content was determined by combustion for 3 h at 550 °C in a muffle furnace. Starch content of the millet grains was determined enzymatically according to ISO15914.

Fungicide analysis was performed on autoclaved, untreated, air-dried wheat straw using gas chromatography/mass spectrometry according to NEN EN 12393.

### Ergosterol

Ergosterol determination of freeze-dried material was based on Niemenmaa et al. [26]. In summary, 200 mg of the material was saponified in 10 % KOH in methanol for 1 h at 80 °C. After cooling, hexane and distilled water were added for extraction. The samples were shaken for 10 min and centrifuged for 15 min at 4,000 rpm. The hexane phase was collected and the hexane-water extraction was repeated once. The hexane phases of the 2 extractions were pooled and evaporated under vacuum. The extracted ergosterol was dissolved in 1 mL methanol before ergosterol content was determined by a high performance liquid chromatography (HPLC) fitted with a reversed phase C18 column (250 × 4.6 mm, Phenomex aqua 5 μm). The liquid phase was 90 % methanol and 10 % (1:1) 2-propanol/hexane. Areas under the peak were corrected for the extraction efficiency based on the internal standard cholecalciferol (vitamin D_3_) (9.6 μg added) (Sigma Aldrich, St. Louis, Missouri, USA), using Empower 2 software (Waters Corporation, Milford, Massachusetts, USA). Mycelium of *C. subvermispora* and *L. edodes* grown on malt extract plates covered with cellophane was freeze dried and subjected to ergosterol extraction. The ergosterol content of the pure mycelium was used to calculate the amount of fungal biomass formed.

### Statistical analysis

The effect of amount of inoculum, particle size and incubation on ergosterol content, detergent fiber composition and IVGP was tested using the generalized linear model (GLM) analysis in SAS software version 9.3 (SAS Institute Inc., Cary, North Carolina, USA). The following model was used:

$$ {\mathrm{Y}}_{\mathrm{i}\mathrm{jk}} = \upmu + {\upalpha}_{\mathrm{i}} + {\upbeta}_{\mathrm{j}} + {\upgamma}_{\mathrm{k}} + {\upomega}_{\mathrm{i}\mathrm{jk}} $$

in which Y_ijk_ is the observation at incubation time i; μ is the overall mean; α_i_ is the fixed effect of amount of inoculum i; β_j_ is the fixed effect of particle size j; γ_k_ is the fixed effect of incubation time k; ω_ijk_ is the random error.

The results of ergosterol measurements, chemical analysis and IVGP at different incubation times of the fungal treatment of each substrate, for each amount of inoculum and particle size combination, were compared using the generalized linear model (GLM) analysis in SAS software version 9.3 (SAS Institute Inc., Cary, North Carolina, USA). Post-hoc multiple comparison with Tukey’s significant test at a level of α = 0.05 was performed to determine the significance of differences between the treatments. The following model was used:

$$ {\mathrm{Y}}_{\mathrm{i}\mathrm{j}} = \upmu + {\upalpha}_{\mathrm{i}} + {\upomega}_{\mathrm{i}\mathrm{j}} $$

in which Y_ij_ is the observation j at incubation time i; μ is the overall mean; α_i_ is the fixed effect of incubation time i; ω_ij_ is the random error.

## Results

Only results of *C. subvermispora* treated wood chips and *L. edodes* treated wheat straw are presented. Visually no or limited growth was observed in *L. edodes* treated wood chips and *C. subvermispora* treated wheat straw. This low growth was caused by unknown external factors.

### Inoculum

The chemical composition and the IVGP of millet used as spawn at the moment of inoculation (5 wk of colonization) are presented in Table 1. Ergosterol contents showed that the millet was colonized by both *C. subvermispora* and *L. edodes*. To estimate the amount of mycelial biomass we have measured the amount of ergosterol per unit dried mycelium for each fungus after growth until full colonization (approximately 10 d) on malt extract plates. *C. subvermispora* contained 9.6 mg ergosterol/g mycelium and *L. edodes* contained 6 mg ergosterol/g mycelium. Each g of colonized millet contains thus approximately 14.3 mg *C. subvermispora* mycelium or 32.1 mg *L. edodes* mycelium at the moment of inoculation. However, it should be noted that the ergosterol contents of pure mycelium grown on malt extract agar may be different for the ergosterol contents of mycelium grown on millet grains. Colonized millet had a lower (*P* < 0.05) ADL and cellulose content than the uninoculated grains (control). Although *L. edodes* numerically decreased the hemicellulose content, this was not significant, whereas *C. subvermispora* significantly decreased the hemicellulose content. Interestingly, the starch content of the *C. subvermispora* spawn was not different from the control, whereas *L. edodes* significantly degraded the starch in the millet grains. The spawn showed a high IVGP of approximately 300 mL/g OM, which suggests that the addition of spawn to the substrates may have contributed to the increase in IVGP at the start of fungal treatment.

**Table 1:** Chemical composition (g/kg DM) of spawn at the moment of inoculation

Treatment	ADL	Hemicellulose	Cellulose	Starch	IVGP	Ergosterol
Control	14.4^a^	54.0^a^	76.3^a^	699.0^a^	319.7	0.0^c^
*C. subvermispora*	2.3^b^	30.3^b^	50.4^b^	680.2^a^	285.8	138.1^b^
*L. edodes*	3.5^b^	40.9^ab^	43.2^b^	408.2^b^	302.5	192.7^a^
SEM	1.58	3.31	2.65	5.56	16	10.61
*P*-value	0.003	0.0068	0.0003	<0.0001	0.3855	<0.0001

Values with different superscripts within column are significantly (*P* < 0.05) different

*ADL* acid detergent lignin, *IVGP* in vitro gas production in rumen fluid

### *C. subvermispora* treatment of wood chips

**Fig. 1:**
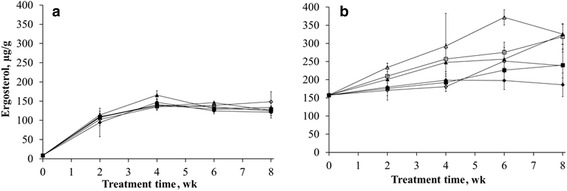
Results of ergosterol measurements. (**a**) *C. subvermispora* on wood chips, (**b**) *L. edodes* on wheat straw. ♦ 0.5% inoculum per g wet substrate (0.5 cm) ■ 1.5 % inoculum per g wet substrate (0.5 cm) ▲ 3 % inoculum per g wet substrate (0.5 cm) ◊ 0.5 % inoculum per g wet substrate (2 cm) □ 1.5 % inoculum per g wet substrate (2 cm) Δ 3 % inoculum per g wet substrate (2 cm)

The probability values of the effects of the amount of inoculum added, particle size and incubation time are shown in Table 2. The amount of inoculum had only a significant effect on the cellulose degradation during the *C. subvermispora* treatment of wood chips. Changing the particle size of wood chips had significant effects on the hemicellulose and cellulose degradation by *C. subvermispora*. No significant effects were observed for the amount of inoculum (content *P* = 0.1435, absolute amounts *P* = 0.4626) and particle size (content *P* = 0.5688, absolute amount *P* = 0.9148) on the ADL degradation. The time of incubation showed effects (*P* < 0.05) on all variables measured. Tables 3 and 4 show that most changes occurred during the first 4 wk of incubation. *C. subvermispora* colonized the wood chips within the first 4 wk of incubation as the ergosterol content increased during the first 4 wk, after which the growth rate reduced (Fig. 1). Only after 4 wk of incubation differences were seen between treatments. Addition of 3 % inoculum to 0.5 cm wood chips resulted in a higher (*P* < 0.05) ergosterol content compared to the other treatments. Only the addition of 3 % inoculum to 0.5 cm wood chips caused a significant decrease (*P* < 0.05) in cellulose content (g/kg DM) and absolute amount of cellulose (g).

**Table 2:** Probability values for effects of the amount of inoculum added (0.5, 1.5 and 3.0 %), particle size (0.5 and 2.0 cm) and incubation time (2, 4, 6 and 8 wk) on chemical composition, ergosterol content and in vitro gas production of two fungal treated substrates (wheat straw and wood chips)

	*C. subvermispora* treated wood chips			*L. edodes* treated wheat straw		
Item	Amount of inoculum	Particle size	Incubation time	Amount of inoculum	Particle size	Incubation time
ADL, g/kg DM	0.1435	0.5688	<0.0001	0.0346	<0.0001	<0.0001
ADL, g	0.4626	0.9148	<0.0001	0.1640	<0.0001	<0.0001
HC, g/kg DM	0.3704	<0.0001	<0.0001	0.0003	<0.0001	<0.0001
HC, g	0.2533	<0.0001	<0.0001	0.0054	<0.0001	<0.0001
Cell, g/kg DM	0.0002	<0.0001	<0.0001	0.0026	0.0003	<0.0001
Cell, g	0.0197	<0.0001	0.0012	0.4314	0.1179	0.0120
Ergosterol, mg/g	0.5085	0.3220	<0.0001	<0.0001	<0.0001	<0.0001
IVGP, mL/g OM	0.4508	0.6735	<0.0001	0.1910	<0.0001	<0.0001

*ADL* acid detergent lignin, *HC* hemicellulose, *Cell* cellulose, *IVGP* in vitro gas production after 72h in rumen fluid

**Table 3:** Changes in detergent fiber content (g/kg DM) over an eight wk period of 0.5 and 2 cm wood chips incubated with three different amounts of *C. subvermispora* inoculum

Particle size		0.5 cm			2 cm		
Inoculum, %		0.5	1.5	3.0	0.5	1.5	3.0
	Wk						
ADL	0	214.8^a^	214.8^a^	214.8^a^	214.8^a^	214.8^a^	214.8^a^
	2	197.8^a^	162.4^b^	162.6^b^	178.3^b^	163.7^b^	156.5^b^
	4	127.0^b^	130.0^c^	126.3^c^	133.2^c^	126.8^c^	134.3^c^
	6	128.3^b^	118.4^d^	123.4^c^	111.8^d^	126.4^c^	126.4^c^
	8	112.0^b^	123.6^cd^	129.4^c^	126.0^c^	121.2^c^	120.7^c^
SEM		9.95	2.07	2.18	2.31	5.41	2.95
*P*-value		<.0001	<.0001	<.0001	<.0001	<.0001	<.0001
HC	0	151.5^a^	151.5^a^	151.5^a^	151.5^a^	151.5^a^	151.5^ab^
	2	154.1^a^	160.7^a^	170.7^a^	151.7^a^	156.2^a^	158.3^a^
	4	90.0^b^	98.5^b^	92.8^b^	109.4^bc^	114.1^b^	129.9^abc^
	6	107.4^b^	85.6^b^	87.0^b^	126.8^ab^	106.2^b^	122.6^bc^
	8	74.5^b^	73.1^b^	74.8^b^	94.9^c^	107.3^b^	112.1^c^
SEM		8.21	10.80	9.82	5.50	4.32	6.55
*P*-value		0.0001	0.0005	0.0001	<.0001	<.0001	0.0025
Cell	0	381.3^a^	381.3^b^	381.3^ab^	381.3^b^	381.3^b^	381.3^b^
	2	390.3^ab^	393.1^ab^	375.3^b^	408.2^b^	396.8^b^	387.4^b^
	4	417.0^ab^	403.3^ab^	384.5^ab^	414.7^ab^	430.3^a^	422.2^a^
	6	417.9^ab^	415.4^ab^	405.4^a^	455.1^a^	446.0^a^	419.3^a^
	8	430.3^a^	419.1^a^	397.1^ab^	424.2^ab^	438.4^a^	419.2^a^
SEM		10.34	7.90	5.98	9.97	6.82	6.42
*P*-value		0.0349	0.0362	0.0301	0.0054	0.0002	0.0019

Values with different superscripts within column are significantly (*P* < 0.05) different. *ADL* acid detergent lignin, *HC* hemicellulose, *Cell* cellulose, *SEM* standard error of the mean

**Table 4:** Changes in absolute amounts of detergent fiber (g) over an eight wk period of 0.5 and 2 cm wood chips incubated with three different amounts of *C. subvermispora* inoculum

Particle size		0.5 cm			2 cm		
Inoculum, %		0.5	1.5	3.0	0.5	1.5	3.0
	Wk						
ADL	0	31.2^a^	31.2^a^	31.2^a^	31.2^a^	31.2^a^	31.2^a^
	2	27.5^a^	22.3^b^	22.4^b^	24.3^b^	23.0^b^	21.3^b^
	4	16.2^b^	16.9^c^	16.3^c^	17.3^c^	16.6^c^	18.1^c^
	6	16.4^b^	14.9^d^	15.8^c^	14.0^d^	16.1^c^	16.5^cd^
	8	13.8^b^	15.1^d^	16.4^c^	15.5^d^	15.5^c^	15.2^d^
SEM		1.60	0.30	0.31	0.35	0.80	0.52
*P*-value		<.0001	<.0001	<.0001	<.0001	<.0001	<.0001
HC	0	22.0^a^	22.0^a^	22.0^a^	22.0^a^	22.0^a^	22.0^a^
	2	21.4^a^	22.1^a^	23.5^a^	20.7^a^	21.9^a^	21.6^ab^
	4	11.5^b^	12.8^b^	12.0^b^	14.2^bc^	14.9^b^	17.5^bc^
	6	13.7^b^	10.8^b^	11.1^b^	15.9^b^	13.6^b^	16.0^c^
	8	9.2^b^	9.0^b^	9.5^b^	11.7^c^	13.7^b^	14.1^c^
SEM		1.19	1.47	1.30	0.83	0.59	0.94
*P*-value		<.0001	0.0001	<.0001	<.0001	<.0001	0.0005
Cell	0	55.5	55.5	55.5^a^	55.5	55.5	55.5
	2	54.1	54.0	51.7^ab^	55.7	55.7	52.7
	4	53.2	52.3	49.8^b^	53.9	56.3	57.0
	6	53.2	52.3	51.9^ab^	57.0	57.0	54.6
	8	52.9	51.3	50.5^b^	52.1	55.9	52.9
SEM		1.59	1.05	0.81	1.25	0.91	0.91
*P*-value		0.7836	0.1072	0.0048	0.133	0.7735	0.0402

Values with different superscripts within column are significantly (*P* < 0.05) different. *ADL* acid detergent lignin, *HC* hemicellulose, *Cell* cellulose, *SEM* standard error of the mean

Generally, in all tested conditions, the ADL and hemicellulose content and their absolute amounts were also decreased during the first 4 wk with *C. subvermispora* incubation. After 4 wk, the lowest values (*P* < 0.05) of hemicellulose and cellulose (contents and absolute amounts) were reached in the treatments with 3 % inoculum added to 2 cm wood chips compared to the other treatments. The lower carbohydrate and unchanged ADL degradation suggest a more selective delignification process when 3 % inoculum was added to 2 cm wood chips. The least selective delignification was observed when 0.5 % inoculum was added to 0.5 cm wood chips, since most hemicellulose and a similar amount of ADL was degraded by *C. subvermispora.* The higher hemicellulose degradation did not result in a higher ergosterol content or a lower IVGP. The IVGP increased (*P* < 0.05) during the first 4 wk of incubation. Most treatments reached a plateau level, whereas addition of 1.5 % inoculum to 0.5 cm wood chips and addition of 3 % inoculum to 2 cm wood chips caused a continuous increase in IVGP. As a result the addition of 1.5 % inoculum to 0.5 cm wood chips resulted in the highest IVGP after 8 wk of *C. subvermispora* incubation.

### *L. edodes* treatment of wheat straw

The significance of the effect of the amount of inoculum, particle size and incubation time are shown in Table 2. The main differences observed in *L. edodes* treatment of wheat straw were seen between particle sizes and incubation times. The particle size of wheat straw had a significant effect on all variables measured, except the absolute amounts of cellulose. The time of incubation had an effect (*P* < 0.05) on all variables measured. The amount of inoculum had a significant effect on the ergosterol content, absolute amounts of hemicellulose and ADL and hemicellulose and cellulose contents.

Addition of 1.5 or 3 % *L. edodes* inoculum to 0.5 cm wheat straw particles resulted in a continuous growth during 8 wk, whereas addition to 2 cm wheat straw particles resulted in growth which stopped after 4 wk of incubation. No changes in ergosterol content were observed throughout the entire incubation period when 0.5 % inoculum was added to 0.5 cm wheat straw particles.

Regardless how much inoculum was added, the ADL content and absolute amounts of 0.5 cm wheat straw did not change after 4 to 6 wk of *L. edodes* incubation (Tables 5 and 6). In contrast, the ADL in 2 cm wheat straw decreased continuously during the 8 wk of incubation (Tables 5 and 6). After 8 wk of incubation, the ADL content of 2 cm wheat straw was lower (*P* < 0.05) than that of the 0.5 cm particles (Tables 5 and 6). The hemicellulose content decreased (*P* < 0.05) during the first 4 to 6 wk (Table 5). The final hemicellulose content and the absolute amounts after 6 and 8 wk of incubation were lower for the 2 cm particles than for the 0.5 cm particles (Tables 5 and 6). The cellulose content increased more (*P* < 0.05) for the 0.5 cm particles than for the 2 cm particles (Table 5). This effect was more visible by the absolute amounts of cellulose, where only a significant decrease in time was seen when 1.5 % inoculum was added to 0.5 cm wheat straw particles (Table 6).

**Table 5:** Changes in detergent fiber content (g/kg DM) over an eight wk period of 0.5 and 2 cm wheat straw incubated with three different amounts of *L. edodes* inoculum

Particle size		0.5 cm			2 cm		
Inoculum, %		0.5	1.5	3.0	0.5	1.5	3.0
	Wk						
ADL	0	90.8^abc^	90.8^ab^	90.8^a^	90.8^b^	90.8^a^	90.8^a^
	2	95.7^a^	100.2^a^	97.9^a^	107.3^a^	97.3^a^	92.6^a^
	4	92.7^ab^	95.4^ab^	82.6^ab^	89.1^b^	79.5^b^	79.3^b^
	6	81.9^bc^	80.5^ab^	95.6^a^	68.2^c^	64.1^c^	64.5^c^
	8	78.6^c^	79.0^b^	69.9^b^	56.5^d^	54.6^d^	52.2^d^
SEM		2.74	4.40	3.82	1.80	1.63	2.36
*P*-value		0.0056	0.0261	0.0025	<.0001	<.0001	<.0001
HC	0	287.1^a^	287.1^a^	287.1^a^	287.1^a^	287.1^a^	287.1^a^
	2	288.8^a^	277.8^a^	262.8^ab^	286.9^a^	273.6^a^	262.1^b^
	4	253.4^b^	260.7^ab^	235.4^bc^	263.7^a^	242.5^b^	233.1^c^
	6	224.7^c^	228.9^bc^	247.2^bc^	211.4^b^	204.8^c^	190.7^d^
	8	240.8^bc^	223.6^c^	221.0^c^	199.9^b^	188.2^c^	196.2^d^
SEM		5.22	7.96	6.04	8.39	4.80	3.44
*P*-value		<.0001	0.0006	0.0002	<.0001	<.0001	<.0001
Cell	0	474.1^b^	474.1^b^	474.1^ab^	474.1	474.1^b^	474.1^bc^
	2	480.4^ab^	470.5^b^	461.1^b^	493.5	475.6^b^	467.1^c^
	4	489.5^ab^	503.1^a^	485.5^a^	481.3	500.3^a^	486.9^abc^
	6	493.8^a^	491.2^ab^	478.7^ab^	500.7	498.3^a^	503.0^a^
	8	489.8^ab^	479.6^ab^	478.1^ab^	508.3	507.4^a^	494.1^ab^
SEM		4.04	6.20	4.63	8.32	4.17	4.62
*P*-value		0.035	0.0221	0.0403	0.0835	0.0005	0.0016

Values with different superscripts within column are significantly (*P* < 0.05) different. *ADL* acid detergent lignin, *HC* hemicellulose, *Cell* cellulose, *SEM* standard error of the mean

**Table 6:** Changes in absolute amounts of detergent fiber (g) over an eight wk period of 0.5 and 2 cm wheat straw incubated with three different amounts of *L. edodes* inoculum

Particle size		0.5 cm			2 cm		
Inoculum, %		0.5	1.5	3	0.5	1.5	3
	Wk						
ADL	0	17.7^a^	17.7^ab^	17.7^a^	17.7^b^	17.7^a^	17.7^a^
	2	18.4^a^	19.5^a^	19.2^a^	20.6^a^	18.8^a^	18.0^a^
	4	17.5^a^	18.3^ab^	16.0^ab^	16.8^b^	15.2^b^	15.2^b^
	6	15.7^ab^	15.2^b^	18.3^a^	12.7^c^	12.0^c^	12.2^c^
	8	14.6^b^	14.7^b^	13.2^b^	10.4^d^	10.1^d^	9.7^d^
SEM		0.60	0.85	0.77	0.33	0.32	0.43
*P*-value		0.0062	0.0113	0.0019	<.0001	<.0001	<.0001
HC	0	56.0^a^	56.0^a^	56.0^a^	56.0^a^	56.0^a^	56.0^a^
	2	55.6^a^	54.0^a^	51.6^ab^	55.2^a^	52.8^a^	51.1^b^
	4	47.9^b^	50.1^ab^	45.6^c^	49.8^a^	46.3^b^	44.7^c^
	6	43.1^c^	43.2^bc^	47.3^bc^	39.3^b^	38.2^c^	36.1^d^
	8	44.8^bc^	41.7^c^	41.8^c^	36.8^b^	34.8^c^	36.4^d^
SEM		0.96	1.49	1.29	1.59	0.92	0.64
*P*-value		<.0001	0.0001	0.0001	<.0001	<.0001	<.0001
Cell	0	92.5	92.5^ab^	92.5	92.5	92.5	92.5
	2	92.5	91.4^b^	90.6	94.9	91.7	91.1
	4	92.6	96.6^a^	94.0	90.9	95.5	93.5
	6	94.7	92.8^ab^	91.6	93.2	92.9	95.1
	8	91.2	89.5^b^	90.3	93.6	93.9	91.7
SEM		1.38	1.03	1.08	1.59	0.83	1.06
*P*-value		0.5141	0.0076	0.1953	0.5183	0.0667	0.1257

Values with different superscripts within column are significantly (*P* < 0.05) different. *ADL* acid detergent lignin, *HC* hemicellulose, *Cell* cellulose, *SEM* standard error of the mean

**Fig. 2:**
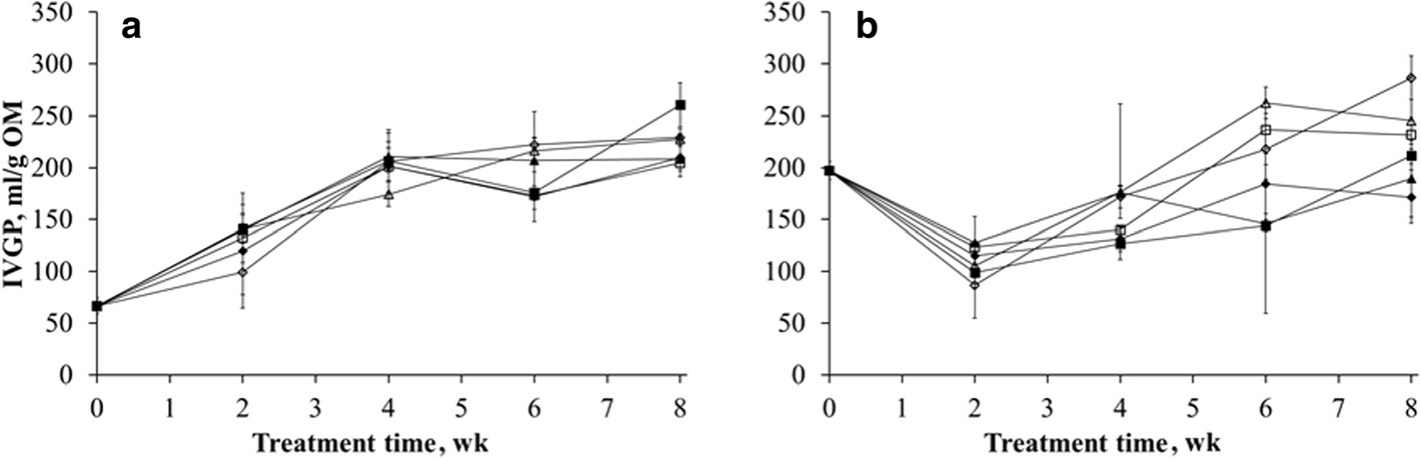
Results of in vitro gas production (IVGP) measurements. (**a**) *C. subvermispora* on wood chips, (**b**) *L. edodes* on wheat straw. ♦ 0.5 % inoculum per g wet substrate (0.5 cm) ■ 1.5 % inoculum per g wet substrate (0.5 cm) ▲ 3 % inoculum per g wet substrate (0.5 cm) ◊ 0.5% inoculum per g wet substrate (2 cm) □ 1.5 % inoculum per g wet substrate (2 cm) Δ 3% inoculum per g wet substrate (2 cm)

Six to eight wk of *L. edodes* treatment increased the IVGP of the 2 cm wheat straw particles more (*P* < 0.05) than that of the 0.5 cm wheat straw particles (Fig. 2b).

## Discussion

The aim of this study was to optimize the colonization conditions of fungal treatments. To do so, the amount of inoculum added and the particle size of the substrate was varied. Here, millet was chosen as inoculation material, because of its small grain size. Addition of the same weight of spawn will result in more inoculation points when using a smaller grain. The IVGP was not different between the different amounts of inoculum added. If the spawn would contribute to IVGP, this would be mainly because millet contains starch, especially in *C. subvermispora* treated biomass since this fungus did not degrade starch upon colonization. The observation that *C. subvermispora* did not utilize starch is interesting as the publically available genome data of *C. subvermispora* shows the presence of genes encoding for amylase. The ergosterol content data show that *C. subvermispora* grew on both millet and wood chips, suggesting millet can be used as spawn for *C. subvermispora*. The data presented here, indicate that hemicellulose and to some extent cellulose are the substrates used by *C. subvermispora* to grow on millet grains. The estimation of the amount of fungal biomass on grains has been done previously for the button mushroom *Agaricus bisporus* using three independent methods including the ergosterol method [27]. These authors calculated that spawn contains circa 10 mg dry mycelial biomass per gram of grain which is in the same range as we have measured here (14 to 32 mg for *C. subvermispora* and *L. edodes* respectively).

*C. subvermispora*, in contrast to *L. edodes*, showed a low growth on wheat straw. In some treatments, such as addition of 0.5 % inoculum to 0.5 cm wheat straw particles, also a low growth of *L. edodes* was found compared to other studies [8, 13]. A possible explanation for the low efficiency of the fungal treatment of wheat straw in the present study is the presence of fungicides. Analysis of the wheat straw showed that it contained 0.767 mg tebuconazole/kg dry substrate. Tebuconazole is a fungicide used in wheat production to control plant pathogenic fungi. The minimal inhibitory concentration of tebuconazole has been shown to be different for different basidiomycete species [28]. Possibly *C. subvermispora* is more sensitive to this fungicide than *L. edodes*. Tebuconazole inhibits the formation of ergosterol in fungi [28]. *C. subvermispora* contains more ergosterol per g of mycelium (9.6 mg ergosterol/g mycelium) than *L. edodes* (6 mg ergosterol/g mycelium), which may be the reason why *C. subvermispora* is more sensitive to this fungicide. Since the use of fungicides is general practice in wheat production, it is advised for future fungal treatments to use organically grown wheat straw. However, *L. edodes* showed a reduced growth on wood chips. A possible explanation may be the different moisture content between both wood chips and wheat straw. The moisture content of wheat straw was around 60 %, while that of wood chips was around 45 %. This difference is inherent to the manner of moisturizing the substrates. After full penetration of the water, the ‘free’ water is removed by draining. The water holding capacity of wood chips is lower than that of wheat straw, causing a difference in moisture content. However there may be a different water activity for both substrates, which was not measured. Possibly *L. edodes* is more sensitive to water activity than *C. subvermispora*.

The particle size of the substrate had only an effect on the *L. edodes* treatment of wheat straw. Sachs et al. [23] showed that *P. chrysosporium* formed more mycelium on the surface of aspen wood chips compared to the inside of the wood chips. Therefore, it is expected that with a decreasing particle size, and thus a larger surface to volume ratio, the substrate is more accessible and more mycelium can be formed. Despite the different surface to volume ratio, particle size did not have a major influence on the *P. chrysosporium* growth patterns [23]. In the present study, particle size had a significant effect on the ergosterol content, although only addition of 0.5 % inoculum to 0.5 cm wheat straw particles had a remarkable low ergosterol content. Probably the contrast between the surface to volume ratios between particle sizes was not large enough to see effects on colonization. Interestingly, 2 cm particles resulted in a lower ADL and a higher IVGP upon the fungal treatment. Gómez [29] demonstrated an increased expression of cellulases and xylanases by *Trametes* sp., when grown on smaller particles of corn straw, compared to larger particles. Carbohydrates in smaller particles are possibly more exposed at the surface compared to large particles. To reach the carbohydrates in large particles, the fungi first have to degrade more lignin. On the other hand, not more carbohydrate degradation was observed in the smaller particles.

It has to be noted that the detergent fiber analysis method was used in this study. The detergent fiber method is developed to estimate feeding value of forage for ruminants. It is not developed to precisely determine the chemically defined fractions, and as a result it underestimates the lignin fraction and overestimates the carbohydrate fractions [30, 31]. Nevertheless, the acid detergent lignin is highly correlated to rumen degradability for both wheat straw and wood chips [13], and changes in the lignin and carbohydrate fractions upon fungal treatment are a good indication for carbohydrate accessibility. For a more detailed chemical analysis of plant cell walls it is advised to measure lignin and carbohydrates in more detail using pyrolysis coupled to gas chromatography and mass spectrometry and sugar analysis.

Particle size did not have an effect on the *C. subvermispora* treatment of wood chips. If a larger contrast between the particle sizes was used, maybe an effect would have been seen. In the scientific literature however, 15 mm size corn stover yielded less glucose upon enzymatic saccharification than 10 and 5 mm corn stover after a *C. subvermispora* treatment [32]. The same authors concluded that moisture content, time and temperature were also important factors influencing the effectiveness of the fungal treatment. Different substrates show different water holding capacities, which also depends on the particle size, explaining why the effect of particle size may be different for different substrates.

The results of this study show that the particle sizes (0.5 or 2 cm) tested do not have a major influence on the *C. subvermispora* treatment of wood chips. Using wheat straw particles smaller than 2 cm does not have beneficial effects on the *L. edodes* treatment.

## Conclusions

The major changes in chemical composition, IVGP and ergosterol content occur during the first 4 wk of *C. subvermispora* treatment of wood chips. The particle size (0.5 or 2 cm) of the wood chips and the amount of inoculum (0.5, 1.5 or 3 %) added do not have significant effects on the *C. subvermispora* treatment. Colonization does not seem to be the limiting factor for *C. subvermispora*. The *L. edodes* treatment of wheat straw was influenced by the particle size of the substrate. Larger (2 cm) particles resulted in a more selective delignification and a higher IVGP than smaller (0.5 cm) particles. The amount of inoculum to some extent affects *L. edodes* treatment of wheat straw. At least 1.5% spawn should be added to obtain a more selective lignin degradation and an increased IVGP.

## Abbreviations

ADF, acid detergent fiber; ADL, acid detergent lignin; Cell, cellulose; DM, dry matter; GLM, generalized linear model; HC, hemicellulose; HPLC, high performance liquid chromatography; IVGP, in vitro gas production; NDF, neutral detergent fiber; OM, organic matter; SEM, standard error of the mean
